# Using Mitrofanoff's principle and Monti's technique as a surgical option for bladder augmentation with a continent stoma: a case report

**DOI:** 10.1186/1752-1947-5-49

**Published:** 2011-02-03

**Authors:** Marcelo F Cassini, Antonio A Rodrigues, Silvio Tucci, Adauto J Cologna, Rodolfo B Reis, Antonio CP Martins, Haylton J Suaid

**Affiliations:** 1 Department of Surgery and Anatomy, Division of Urology, Ribeirao Preto Medical School, Sao Paulo University, Sao Paulo, Brazil

## Abstract

**Introduction:**

Hydronephrosis, reflux and renal failure are serious complications that occur in patients with neurogenic bladder associated with myelomeningocele. When the bladder compliance is lost, it is imperative to carry out surgery aimed at reducing bladder storage pressure. An ileocystoplasty, and for patients not suitable for intermittent catheterization, using the Mitrofanoff principle to form a continent stoma and the subsequent closure of the bladder neck, can be used. We report here, for the first time to the best of our knowledge, an association between two previously described techniques (the Mitrofanoff principle and the technique of Monti), that can solve the problem of a short appendix in obese patients.

**Case presentation:**

A 33-year-old male Caucasian patient with myelomeningocele and neurogenic bladder developed low bladder compliance (4.0 mL/cm H_2_O) while still maintaining normal renal function. A bladder augmentation (ileocystoplasty) with continent derivation principle (Mitrofanoff) was performed. During surgery, we found that the patient's appendix was too short and was insufficient to reach the skin. We decided to make an association between the Mitrofanoff conduit and the ileal technique of Monti, through which we performed an anastomosis of the distal stump of the appendix to the bladder (with an antireflux valve). Later, the proximal stump of the appendix was anastomosed to an ileal segment of 2.0 cm that was open longitudinally and reconfigured transversally (Monti technique), modeled by a 12-Fr urethral catheter, and finally, the distal stump was sutured at the patient's navel.

After the procedure, a suprapubic cystostomy (22 Fr) and a Foley catheter (10 Fr) through the continent conduit were left in place. The patient had recovered well and was discharged on the tenth day after surgery. He remained with the Foley catheter (through the conduit) for 21 days and cystostomy for 30 days. Six months after surgery he was continent with good bladder compliance without reflux and fully adapted to catheterization through the navel.

**Conclusion:**

The unpublished association between the Mitrofanoff and Monti techniques is feasible and a very useful alternative in urologic cases of derivation continent in which the ileocecal appendix is too short to reach the skin (i.e., in obese patients).

## Introduction

The development of hydronephrosis, vesicoureteral reflux and renal scarring with subsequent progression to renal failure are feared and serious complications that can occur in patients with neurogenic bladder associated with myelomeningocele. Such patients should be treated starting in childhood with clean and intermittent catheterization along with anticholinergics in the presence of detrusor overactivity. In severe cases (i.e., sphincter dyssynergia), there are greater risks of renal failure. When irrespective of the treatment, a patient has an unsatisfactory development and loss of bladder compliance, it is imperative to avoid future renal failure by surgeries aimed at reducing bladder storage pressure. Our service routinely uses ileocystoplasty and, for patients unable or not adapted to intermittent catheterization, Mitrofanoff's principle (in which the vascularized ileocecal appendix is anastomosed at the skin and bladder, with a nonrefluxing valve and continent stoma). We then proceed with closure of the bladder neck.

## Case Presentation

A 33-year-old male Caucasian patient with myelomeningocele and neurogenic bladder had an unsatisfactory clinical progression despite treatment with intermittent catheterization and anticholinergics. Not suitable for catheterization, the patient developed low bladder compliance (4.0 mL/cm H_2_O) and grade II left vesicoureteral reflux. Although his renal function was still preserved, bladder augmentation (ileocystoplasty) was indicated.

**Figure 1:**
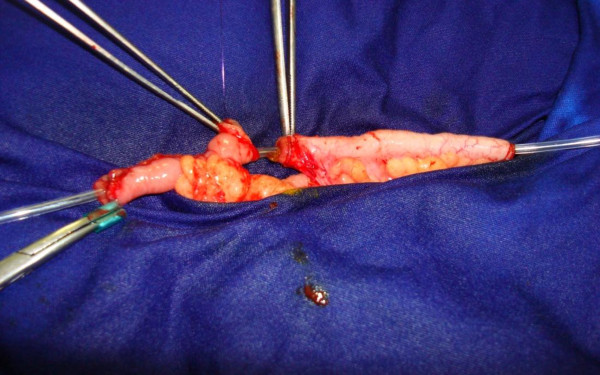
**Association of Mitrofanoff's principle and Monti's technique**. Anastomosis of vascularized appendix (*right*) and ileal conduit (*left*).

We used 15 cm of ileum, 20 cm from the ileocecal valve, that was opened longitudinally and reconfigured into a "U" shape, with continent derivation (Mitrofanoff's principle). During surgery, we found a short ileocecal appendix that was insufficient to reach the skin. Then it was decided to make an association between the Mitrofanoff's principle and the Monti's technique of ileal conduit by which we performed an anastomosis of the distal stump of the appendix to the bladder with an antireflux valve. Then, the proximal stump of the appendix was anastomosed to an ileal segment of 2.0 cm that was open longitudinally and reconfigured transversally (Monti technique), modeled by a 12-Fr urethral catheter, and finally, the distal stump was sutured at the patient's navel (Figure 1). After the procedure, we left in place a suprapubic cystostomy (22 Fr) and a Foley catheter (12 Fr) through the continent conduit after bladder neck closure in two layers using 2.0 Vicryl. The patient recovered well, restarting an early progressive diet and ambulation. He stayed in the hospital for 10 days after surgery, and the Foley catheter remained for 30 days (through conduit) and cystostomy by 45 days. At present, the patient is continent with good vesical augmentation compliance (35-50 mL/cm H_2_O) and no urethral reflux, and he is well adapted to intermittent catheterization of the umbilicus.

## Discussion

The Mitrofanoff principle for creation of a continent catheterizable stoma using the appendix has been a mainstay in the armamentarium of pediatric urologists and reconstructive surgeons since it was originally described in 1980 [1]. This principle involves the use of a small-caliber tube implanted into a compliant bladder or reservoir with a nonrefluxing anastomosis to provide a convenient and effective method of emptying the bladder. Applications of this technique have expanded to include the use of ureter, tapered ileum, stomach, tubularized bladder flaps, and transverse tubularized bowel, as originally described by Yang [2] and Monti et al [3] and later modified by Casale [4]. Interest in these other tissues stems from the occasional lack of a suitable appendix or, more commonly, the desire to use the appendix for the Malone antegrade continent enema (MACE) procedure [5]. Alternatives such as the "double Monti" technique (with two Yang-Monti channels in a line) can also be used. Overall, we have achieved better results in urinary continence when we performed the antireflux valve with the appendix than the ileum (Monti). The lack of a suitable appendix may be a more common problem in adults because the thickness of the abdominal wall and the pelvic position of the bladder can make the distance between the skin and bladder too long to create a tension-free anastomosis. This result is true if the umbilicus is chosen for the stoma. Especially in this case, the adipose tissue of the patient's abdomen was very thick, and the appendix was too short to be used directly and reach the skin at the right iliac fossa. Another relevant factor is that the navel's skin, naturally already through the abdominal wall, facilitates the anastomosis with the continent conduit, making the patient's abdomen more aesthetically pleasing.

An interesting option to solve this problem is related here. A 2- to 3-cm segment of bowel (usually ileum) is isolated on its mesentery in the standard fashion. If simultaneous augmentation or a neobladder creation is being performed, the Monti segment should be harvested from an adjacent section, eliminating the need for an additional bowel anastomosis (see Figure 1). The segment is then opened longitudinally along the antimesenteric border and retubularized transversely with absorbable suture over a 12-Fr catheter. Stay sutures placed on both ends of the tube facilitate closure by maintaining tension. The central portion is closed with a running suture, and the ends are closed with an interrupted suture so adjustments to the length of the channel at the site of skin (umbilicus) or appendix and bladder implantation can be made without disrupting the suture line. An added benefit of the procedure is the ability to change the position of the mesentery along the channel by simply changing the location of the initial longitudinal incision. For instance, an asymmetrically positioned mesentery is helpful in patients with a large amount of subcutaneous tissue because channel length is required to reach the skin and the appendix that is anastomosed at the bladder wall with a nonrefluxing anastomosis.

## Conclusion

The association of Mitrofanoff's principle with the Monti procedure is feasible and can be a useful alternative, especially in obese patients who need a continent stoma (reservoir) and who have an appendix that is too small to reach the skin.

## Consent

Written informed consent was obtained from the patient for publication of this case report and accompanying images. A copy of the written consent is available for review by the Editor-in-Chief of this journal.

## Competing interests

The authors declare that they have no competing interests.

## Authors' contributions

MFC wrote the article and was the surgeon of the case. AAR had the idea and helped with the surgery. ST, AJC and ACPM performed the literature review. RBR wrote the discussion and literature review. HJS discussed the case and revised the article. All authors have read and approved the final manuscript.
